# Ultrastrong graphene sheets induced by nanoconfined water

**DOI:** 10.1093/nsr/nwae164

**Published:** 2024-05-11

**Authors:** Jacob Klein

**Affiliations:** Weizmann Institute of Science, Israel

Water and other solvents may exhibit unusual thermodynamic and kinetic properties when nano-confined, as demonstrated in several previous reports [1–4]. For example, extreme cylindrical nanoconfinement has been realized in carbon nanotubes [1], improving ethanol production by an order of magnitude, while the anomalous fluidity of nanoconfined water relative to nanoconfined organic solvents is important for lubrication [2,3]. Nanoconfinement effects for solvent molecules have, moreover, been applied to catalysis, energy storage and other applications [4]. Previous investigations of nanoconfinement effects mainly focused on the physical and chemical properties of the solvent molecules; however, exploiting such nanoconfinement for modulating materials' properties has rarely been attempted.

**Figure 1.**
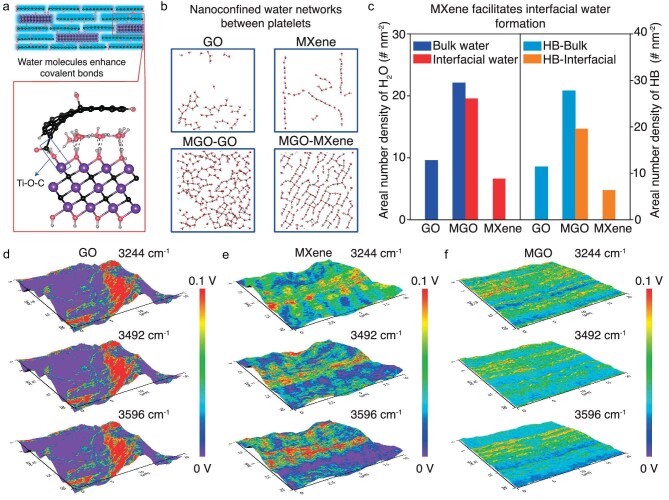
(a) Schematic illustration of the MXene-bridged GO (MGO) sheet. The enlarged image depicts the density functional theory (DFT) calculation–derived atomic structures of MXene-bridged GO in MGO sheets, showing the bridging of GO and MXene nanoplatelets through Ti–O–C covalent bonds. (b) The configuration of water and hydrogen bonds in GO, MXene, and MGO sheets obtained from molecular dynamics calculations. (c) The areal number density of water molecules and hydrogen bonds in GO, MXene, and MGO sheets. (d–f) AFM-IR chemical maps of GO, MXene, and MGO sheets obtained using laser irradiation at 3244, 3492, and 3596 cm^−1^, respectively. Reproduced from ref. [5] with permission.

In a recent pioneering study published in *Science* [5], Prof. Qunfeng Cheng's group at Beihang University found that interlayer nanoconfined water in transition-metal carbides and/or nitrides (MXenes) and graphene oxide (GO) nanoplatelets, can strongly improve their alignment. In this work, MXene and GO nanoplatelets with similar lateral sizes were stirred in water at room temperature to form MXene-bridged GO (MGO) nanoplatelets. These were then assembled into MGO sheets by continued vacuum-assisted filtration. This process could confine almost twice as much water (∼35 wt %) in the nanoplatelets interlayers than in traditional vacuum-assisted filtration. Importantly, as shown in Fig. 1a, the interlayer nanoconfined water could not only facilitate the formation of the Ti–O–C covalent bonds, but also align the nanoplatelets.

Molecular dynamics (MD) simulations revealed the configurations of water molecules and hydrogen bonding (HB) in GO, MXene, and MGO sheets (Fig. 1b). The water molecules in the MXene sheets show an ordered chain-like distribution, whereas the water molecules in the GO sheets show a disordered distribution. In addition, the water on the MXene side of MGO sheets exhibits a more ordered and denser arrangement compared to the GO side (Fig. 1b). The presence of MXene was the main reason why a large amount of nanoconfined water was able to stabilize in the interlayers (Fig. 1c) thus there was only bulk water in the GO sheets and only interfacial water in the MXene sheets, i.e. MXene nanoplatelets in MGO sheets facilitated interfacial water formation in the interlayers. Compared with the bulk water, interfacial water had higher density and stability due to the hydrogen bonds between water molecules and MXene nanoplatelets, thus preventing capillary contraction; this dramatically reduces wrinkling during the assembly process, and greatly improves the composite mechanical properties. The Herman orientation factor of the MGO sheet was 0.87, much higher than that of the GO sheet (0.79). Atomic force microscopy-infrared (AFM-IR) spectroscopy further confirmed the water molecules aggregation and distribution states (Fig. 1d–f). These results showed that the interlayer nanoconfined water could modify the alignment of nanoplatelets, a novel exploitation of nanoconfinement effects.

In sum, Cheng and co-workers [5] provide an important first demonstration of how nanoconfined water may be exploited in assembling high-performance nanocomposites. The resulting greatly-enhanced mechanical properties of the composites hold strong potential for improved energy-storage devices, among others. This innovative strategy may point the way more generally for exploiting liquid-nanoconfinement effects for better materials' properties in a range of applications.
